# Effect on breastfeeding practices of providing in-home lactation support to vulnerable women through the Canada Prenatal Nutrition Program: protocol for a pre/post intervention study

**DOI:** 10.1186/s13006-021-00396-y

**Published:** 2021-07-02

**Authors:** Alison Mildon, Jane Francis, Stacia Stewart, Bronwyn Underhill, Yi Man Ng, Elle Richards, Christina Rousseau, Erica Di Ruggiero, Cindy-Lee Dennis, Deborah L. O’Connor, Daniel W. Sellen

**Affiliations:** 1 Nutritional Sciences, Temerty Faculty of Medicine, University of Toronto, Toronto, Ontario Canada; 2 Translational Medicine Program, The Hospital for Sick Children, Toronto, Ontario Canada; 3 Health Promotion and Community Engagement, Parkdale-Queen West Community Health Centre, Toronto, Ontario Canada; 4 The Stop Community Food Centre, Toronto, Ontario Canada; 5 Tamarack Institute, Waterloo, Ontario Canada; 6 Dalla Lana School of Public Health, University of Toronto, Toronto, Ontario Canada; 7 Lawrence-Bloomberg Faculty of Nursing, University of Toronto, Toronto, Ontario Canada; 8 Anthropology, Faculty of Arts and Sciences, University of Toronto, Toronto, Ontario Canada

**Keywords:** Exclusive breastfeeding, Vulnerable women, Lactation support, Canada Prenatal Nutrition Program

## Abstract

**Background:**

Only one-third of Canadian infants are exclusively breastfed for the first 6 months of life as recommended. Skilled lactation support in the early postpartum period is one strategy for improving breastfeeding outcomes by building breastfeeding self-efficacy and resolving difficulties. Access to such support is limited among vulnerable women, including those who are new immigrants, low income, under-educated, young or single. The Canada Prenatal Nutrition Program (CPNP) aims to improve birth and breastfeeding outcomes among vulnerable women, but currently lacks a formal framework for providing postpartum lactation support.

**Methods:**

This pre/post intervention study will examine the effect on breastfeeding outcomes of an evidence-based in-home lactation support intervention provided through the CPNP. We will enrol 210 pregnant women who intend to breastfeed and are registered CPNP clients at two sites in Toronto, Canada. During the intervention phase, postpartum home visits by International Board Certified Lactation Consultants (IBCLCs) will be pro-actively offered to registered clients of the two sites. Double-electric breast pumps will also be provided to those who meet specific criteria. Infant feeding data will be collected prospectively at seven time points from 2 weeks to 6 months postpartum. Descriptive and regression analyses will be conducted to measure intervention effects. The primary outcome is exclusive breastfeeding at 4 months postpartum. Secondary outcomes include the duration of any and exclusive breastfeeding, timing of introduction of breastmilk substitutes and timing of introduction of solid foods. Breastfeeding self-efficacy will be assessed prenatally and at 2 weeks and 2 months postpartum. Other measures include maternal socio-demographics, infant feeding intentions, maternal depression and anxiety, and household food insecurity. Monitoring data will be used to assess the reach, uptake and fidelity of intervention delivery.

**Discussion:**

Increasing access to skilled lactation support through the CPNP may be an effective means of improving breastfeeding practices among vulnerable women and thereby enhancing health and development outcomes for their infants. This pre/post intervention study will contribute evidence on both the effectiveness and feasibility of this approach, in order to guide the development and further testing of appropriate models of integrating lactation support into the CPNP.

**Trial registration:**

ClinicalTrials.gov (NCT03589963) registered July 18, 2018.

## Background

Breastmilk is a unique food tailored to meet the nutritional, immunological and developmental needs of human infants [1]. The health benefits of breastfeeding are realized in a dose-response relationship to the degree of exclusive breastfeeding, particularly in early infancy [1]. The World Health Organization and Health Canada recommend that all infants be exclusively breastfed (i.e. receive only breastmilk and essential vitamins, minerals and medicines) for the first 6 months of life [2, 3]. However, achievement of this goal remains elusive. Globally, only 42% of infants under 6 months of age are exclusively breastfed [4]. National data show that 90% of Canadian mothers initiate breastfeeding, but nearly half discontinue within the first 6 months and only 32% exclusively breastfeed for 6 months [5]. Exclusive breastfeeding is compromised by a variety of practices, particularly formula supplementation and early introduction of solids [6, 7]. Exclusive breastfeeding duration is lower among vulnerable women in Canada, such as those with lower income and education levels, adolescents, single parents and those living in food insecure households [8–12]. Global systematic review evidence indicates that immigrant women are more likely to initiate and continue breastfeeding than their native-born counterparts, likely reflecting social norms regarding breastfeeding in many countries of origin, but exclusive breastfeeding is a universal challenge [13].

Two key contributors to both early cessation and non-exclusive breastfeeding are the perception of insufficient breastmilk supply and unresolved breastfeeding difficulties [14–17]. Access to skilled lactation support in the early postpartum period can be vital to addressing these concerns and supporting women to achieve their breastfeeding goals [18]. Underlying these concerns is breastfeeding self-efficacy, a construct drawn from Bandura’s social-cognitive theory and defined as a woman’s confidence in her ability to breastfeed her new baby [19]. Women with high breastfeeding self-efficacy are better able to overcome common challenges and continue breastfeeding [19]. Theory-based postpartum lactation support interventions have been shown to improve breastfeeding self-efficacy, duration and exclusivity [20, 21].

Breastfeeding self-efficacy is modifiable through interventions which increase women’s experience of four determinants, the most critical of which is *performance mastery*, or successful achievement of breastfeeding skills [19]. For women who are new to breastfeeding or experiencing difficulties, skilled lactation support in the early postpartum period helps build breastfeeding skills and resolve challenges, directly contributing to performance mastery. Skilled lactation support also contributes to breastfeeding self-efficacy through *verbal persuasion* (positive, confidence-building messages) and *physiological and affective states,* through the mitigation of stress, anxiety and pain related to breastfeeding [19]. The fourth determinant of breastfeeding self-efficacy is *vicarious learning* (observing others successfully breastfeeding), which can be achieved through peer support, media and other forms of role modeling.

Several recent systematic reviews have found evidence of the effectiveness of postpartum skilled lactation support interventions for increasing breastfeeding duration and exclusivity in the first 6 months postpartum [22–25]. The authors of a Cochrane review concluded that postpartum lactation support is likely to be more effective when breastfeeding initiation rates are high, and when the support is proactive and predictable with face-to-face contact [24]. However, access to skilled lactation support which follows these best practice guidelines is limited as many services, such as drop-in breastfeeding clinics and telephone hotlines, are provided reactively after problems occur. Vulnerable women in particular may face barriers related to time, transportation and language skills, which make it difficult for them to access these services [26]. Many studies included in the Cochrane review were conducted with low-income women but sub-group analysis by maternal characteristics was not performed [24].

The Canada Prenatal Nutrition Program (CPNP) is an initiative of the federal government implemented through community agencies with the aim of improving birth outcomes and promoting breastfeeding among vulnerable women [27]. Service provision may extend into the postpartum period but this varies between CPNP sites, with national evaluation data showing that postpartum women constitute only 12% of all clients served [28]. Core CPNP services include prenatal breastfeeding promotion and education. Provision of postpartum breastfeeding support is encouraged [29], but there is currently no formal framework or funding for integrating this support within the CPNP. Strengthening this program component may be a means to increase vulnerable women’s access to skilled lactation support. An example of this is the infant feeding program implemented with additional charitable funds from The Sprott Foundation by the Parkdale Parents’ Primary Prevention Project (5Ps), a CPNP site in Toronto, Canada. Clients of the 5Ps CPNP receive a ‘welcome package’ of infant care and breastfeeding supplies at the time of delivery, and are offered postpartum in-hospital or in-home visits by International Board Certified Lactation Consultants (IBCLCs) and a double-electric breast pump. A qualitative evaluation found that these lactation services addressed key physical, practical and self-efficacy breastfeeding challenges women encountered, including pain, low milk supply, time pressures and stress [26]. Participants highly valued three specific program elements: the provision of *in-home* services delivered by *skilled* providers who were *non-judgemental* [26]. These findings demonstrate the value of integrating postpartum lactation support with the CPNP, but no data are available to determine the effects of the support provided on breastfeeding practices of 5Ps clients.

Drawing on the evidence from breastfeeding self-efficacy theory, systematic reviews and the 5Ps qualitative evaluation, the aims of the current research are to examine the:

i) effects of providing access to in-home postpartum lactation support on breastfeeding practices among women enrolled in the CPNP at two sites in Toronto; and
ii) feasibility of this delivery model as a means to increase vulnerable women’s access to skilled lactation support.

## Methods

### Study design and hypothesis

We will conduct a pre/post intervention study with clients of two CPNP sites in Toronto. Prospectively collected data on infant feeding practices over the first 6 months and breastfeeding self-efficacy scores will be compared between participants who give birth before and after initiation of the lactation support intervention. This quasi-experimental design was chosen as it would not be either ethical or feasible to randomize clients at these community programs targeting vulnerable women to intervention and control groups.

The primary outcome is exclusive breastfeeding at 4 months postpartum. We hypothesize that CPNP clients with access to our lactation support intervention will be more likely to exclusively breastfeed their infants at 4 months postpartum compared with those in the pre-intervention group, and that this effect will be mediated by higher breastfeeding self-efficacy scores. Secondary outcomes are duration of any breastfeeding; duration of exclusive breastfeeding; timing of introduction of breastmilk substitutes (formula); and timing of introduction of solids. We will also assess the rates of any and exclusive breastfeeding and use of expressed breastmilk at each data collection time point up to 6 months postpartum.

Although 6 months is the recommended duration of exclusive breastfeeding, we chose 4 months for the primary outcome analysis as we anticipate that our intervention will improve exclusive breastfeeding primarily by building breastfeeding skills and addressing difficulties in the early postpartum period. Exclusive breastfeeding is frequently compromised after 4 months through early introduction of solids [6, 9], but this practice is less amenable to influence by lactation support provided earlier in the postpartum period.

Monitoring data will be collected and analyzed to determine the reach, uptake and fidelity of the intervention.

### Setting

This research will be embedded within two CPNP sites in Toronto, Ontario: the Great Start Together (GST) program implemented by Parkdale-Queen West Community Health Centre and the Healthy Beginnings (HB) program implemented by The Stop Community Food Centre. These sites were chosen because of their relatively large enrollment size, continuity of service into the postpartum period but limited focus on lactation support, and geographic catchment areas which collectively cover a significant portion of South West Toronto. Both programs operate as weekly drop-in services providing: (1) group workshops; (2) individual support from St. Stephen’s Community House settlement staff and Toronto Public Health nurses and dietitians; (3) referrals to other community services, including doula support; (4) CAD$10 grocery gift card; (5) snacks during service use; (6) two public transit tokens to attend the program; and (7) on-site childcare during service use. Interpreters attend both programs to enable participation of non-English speaking clients. The nurses deliver group workshops on breastfeeding benefits and techniques approximately every 6 weeks and provide limited individual breastfeeding support to clients on request during the program hours. This support is valuable but has limited reach during the critical early postpartum period as many clients do not return to the program for several weeks after giving birth.

Private donations to The Stop Community Food Centre enable the provision of additional supports to HB clients at the weekly program. These include a hot cooked lunch, CAD$5 worth of fresh fruits and vegetables and a food bank hamper typically consisting of 1 L milk, 4 eggs, 2 cups of rice and 2 cups (or 1 can) of beans. HB’s Family Support program provides one-to-one support for the most vulnerable clients through home visits.

Monitoring data show that the majority of clients in the programs are newcomers to Canada, defined as arriving within the past 5 years. In 2017, 222 women registered in the GST program, 67% of whom were newcomers, primarily from China. Chinese interpreter services are provided at the weekly program. From September 2018 to August 2019, 225 women participated in HB, including 134 new clients, 106 of whom were newcomers. In a 2019 internal survey of HB clients (*n* = 92), 55% reported an annual income below the Statistics Canada household-size-adjusted low-income cut-off [30]. Over 50% of HB clients surveyed used interpreter services at the weekly program, which in 2019 included Spanish, Portuguese and American Sign Language.

### Sample size

The sample size estimate is based on the primary outcome. Preliminary data indicated that 53% of program clients were exclusively breastfeeding at 4 months postpartum. Based on an anticipated increase to 73% in the post-intervention group, we require 93 women per group in order to achieve 0.80 power with alpha = 0.05. To allow for 10% attrition we aim to recruit 105 women per group for a total of 210 study participants. The anticipated attrition rate is based on preliminary evidence of high retention in a separate prospective infant feeding study conducted by our group at another CPNP site.

### Recruitment

Eligibility for the study is defined as registration in GST or HB with a due date within the data collection period; intention to try breastfeeding; and intention to continue living in Toronto with the infant for the first 6 months postpartum. Exclusion criteria are preterm birth before 34 weeks gestation; congenital abnormality or medical condition affecting feeding; and hospitalization of the mother or infant at 2 weeks postpartum.

Recruitment will follow a two-step consent process. New clients of the GST or HB complete a program registration form which includes a yes/no question asking for consent to be contacted about the research. The first author (AM) attends the GST and HB programs weekly and will monitor the registration lists to identify clients in their third trimester of pregnancy. Those who have given permission for contact will be approached to explain the study and obtain informed consent. This contact will take place in person if the client attends programming, or by telephone with provision of the consent form by email.

### Intervention

The core component of the intervention is in-home lactation support delivered by IBCLCs, with provision of breast pumps by the IBCLCs to clients meeting specific criteria (described below) as a secondary component. Enrollment in the research study will not be a pre-condition for accessing the intervention. This is both for ethical reasons and to allow assessment of the feasibility of this intervention model, including the demand for lactation services by GST and HB clients. The implementation process was formulated in collaboration with the 5Ps Program Coordinator (co-author SS) and infant feeding program staff in order to benefit from their experience and networks. The implementation procedures align as closely as possible with delivery of other services through the GST and HB programs, but clients will be informed that the intervention is being offered as part of an infant feeding research project.

### Intervention description

Clients registered prenatally in either the GST or HB program who give birth during the intervention period and live in the city of Toronto will have access to in-home lactation support from an IBCLC. This service will be offered pro-actively in the early postpartum period, but will be available throughout the first 6 months. A Research Coordinator will oversee intervention delivery, and professional interpreter services (in-person or by telephone) will be used for communication with non-English speaking women.

The Research Coordinator will attend both weekly programs and approach eligible prenatal clients individually to explain the service, provide her contact details, and record the contact information of clients who are interested. Clients will be encouraged to contact the Coordinator if they need lactation support, but the Coordinator will also pro-actively telephone interested clients around the time of birth to offer the IBCLC support and make the arrangements with the IBCLCs as required. IBCLC visits will take place within 48 h of request by a client, and we anticipate that the majority of initial visits will occur within the first 2 weeks postpartum. Three self-employed IBCLCs already contracted by the 5Ps program have been recruited to deliver the intervention. Private IBCLC services are not covered by provincial health insurance in Ontario, so women who use this resource are typically of higher socio-economic status. The 5Ps staff previously screened many self-employed IBCLCs to select those who practice with a client-centred approach and are able to adapt to the circumstances of vulnerable women, such as communicating through interpreters and visiting low-income homes.

The first home visit will last approximately 1.5 h and include assessment, education and management of the client’s lactation concerns. In-person or telephone interpreters will be provided for clients with limited English fluency. The IBCLC conducting the visit will provide her contact information to the client for follow up contact by text, phone or email. Following the first home visit, the Research Coordinator will contact the client to see if they were satisfied with the service and arrange a second visit with the IBCLC if needed. In cases with more complex lactation issues, a third visit may be offered. Follow up visits are expected to be approximately 1 h in length.

The 5Ps infant feeding program supports include provision of double-electric breast pumps through the IBCLCs, and a majority of clients (70%) receive this instrumental support. Participants in the 5Ps qualitative evaluation with 46 vulnerable women identified that the breast pumps assisted with building or maintaining breastmilk supply, relieving engorgement, allowing others to feed the baby, and providing breastmilk for infants with latching difficulties [26]. Studies in Canada and other high-income countries report widespread use of breast pumps across socio-demographic groups [6, 31, 32]. Current evidence on the association between breast pump use and breastfeeding outcomes is mixed, and individualized guidance on breast pump use is recommended [33, 34]. Our intervention therefore includes breast pump provision as a secondary component. The IBCLCs will provide a double-electric breast pump (Ameda Finesse™) to clients who meet the following criteria: (1) infant unable to latch; (2) mother returning to work or school; or (3) high dependence on infant formula.

In addition to the home visits, the IBCLCs will provide three group workshops in each participating CPNP during the intervention phase of the study. At these workshops the IBCLCs will deliver education regarding the onset of lactation in the neonatal period and tips for successfully establishing breastfeeding, as well as explaining the role of IBCLCs and what to expect in a home visit. The workshops will also provide an opportunity for the Research Coordinator to formally introduce and explain the lactation support intervention.

### Data collection

Data will be collected in person or by telephone either by AM or by a Mandarin speaking Research Assistant. Professional interpreter services will be used as required for other non-English speaking participants.

All primary and secondary outcome measures will be assessed through prospective data collection using a standardized and validated Infant Feeding Questionnaire developed by DLO’s research group and used in several prior research studies at the Hospital for Sick Children in Toronto [35]. This questionnaire will be administered to study participants at 2 weeks postpartum, and then monthly until 6 months for a total of seven infant feeding data collection points (Table 1). At each time point, participants will be asked to report the average number of daily milk feeds their infant received in the past 2 weeks, divided into feeds at the breast, expressed breastmilk feeds, and formula feeds. Provision of cow’s milk, non-milk fluids and vitamin or mineral supplements will also be recorded. Participants who stop breastfeeding will be asked for the last date breastmilk was provided and the main reasons for cessation. If applicable, the date of solids introduction and the name of the first food will be recorded.

**Table 1:** Data collection timeline

Study Instrument	Prenatal	Postnatal Data Collection						
		2 weeks	1 month	2 months	3 months	4 months	5 months	6 months
Maternal Socio-demographics Questionnaire	√							
Infant Feeding Intentions Scale	√							
Prenatal Breastfeeding Self-Efficacy Scale	√							
Household Food Security Questionnaire	√							√
Breastfeeding Self-Efficacy Scale-Short Form		√		√				
Infant Feeding Questionnaire		√	√	√	√	√	√	√
Edinburgh Postnatal Depression Scale				√				
State-Trait Anxiety Inventory for Adults				√				
Six Months Postpartum Questionnaire								√

We hypothesize that the intervention effects will be mediated through increases in breastfeeding self-efficacy, which will be assessed at 2 weeks and 2 months postpartum using the Breastfeeding Self-Efficacy Scale-Short Form [36]. Additional data collection tools are listed below. The Socio-demographics and 6 Months Postpartum questionnaires were developed for this study, with income-related questions drawn from Statistics Canada’s Employment Insurance Coverage Survey [37]. The remaining instruments are validated scales from the literature, all of which have been used previously with vulnerable populations.

- Maternal Socio-demographics Questionnaire – assesses maternal age, education level, parity, ethnicity and for participants not born in Canada, country of origin and length of time in Canada
- Infant Feeding Intentions [38] - validated 5-item scale assessing prenatal intention to exclusively breastfeed for 6 months
- Prenatal Breastfeeding Self-Efficacy Scale [39] – validated 20-item scale; higher prenatal self-efficacy scores are correlated with intention to breastfeed
- Household Food Security Survey Module of the Canadian Community Health Survey [40] – validated 18-item questionnaire; household food insecurity is categorized as marginal, moderate, severe or food secure based on number of affirmative responses
- Edinburgh Postnatal Depression Scale (EPDS) [41] – validated 10-item scale, widely used in perinatal research and clinical practice to assess maternal depressive symptomatology
- State-Trait Anxiety Inventory (STAI) [42] – validated 40-item scale assessing both current state of anxiety (20 items) and tendency or trait anxiety (20 items)
- Six Months Postpartum Questionnaire – collects data on sources of infant feeding advice, participation in other relevant parenting support programmes, adequacy and main source of household financial resources since the infant’s birth, and maternal receipt of federal Employment Insurance maternity benefits (yes/no)

Table 1 shows the timing of data collection for each study instrument. Prenatal data will be collected in the third trimester. The 2-week and 1-month data will be collected within 7 days before or after the exact time point, and the other data will be collected within 14 days before or after the exact time point with the exception of the two maternal mental health forms (EPDS and STAI). These may be collected up to 3 months postpartum. The greater time window is to enable completion of these forms in person due to the sensitive nature of the content and the higher rates of depression and anxiety among newcomer mothers reported in the literature [43]. Research staff will arrange to meet study participants at their home or a preferred location in their community if they are unable to attend either the HB or GST program to complete these forms. A safety protocol will be followed to ensure appropriate support and follow-up for any participants with scores > 21 on the EPDS, or who indicate any thoughts on self-harm in response to EPDS question 10.

The Infant Feeding Questionnaire has been professionally translated into Chinese and back-translated by a second translator. We will utilize published Spanish translations of the Infant Feeding Intentions [38] and Prenatal Breastfeeding Self-Efficacy Scale [44]. Translations into Chinese, Spanish and Portuguese were accessed from a compilation of validation studies for the EPDS [45] and were provided by CLD for the Breastfeeding Self-Efficacy Scale-Short Form and by MindGarden, Inc. for the STAI. Other study forms have been translated into Chinese by our Research Assistant or will be orally translated at the time of data collection by professional interpreters.

In order to monitor feasibility of implementation delivery, the Research Coordinator will track the number of clients eligible for the intervention, the number who participate (defined as receiving at least one IBCLC visit), and the specific services provided (number of home visits and breast pump provision, if applicable). Reasons for clients not accessing the intervention will also be recorded. The IBCLCs will complete a data collection form after each home visit, recording the assessment components covered, concerns identified and services provided. These data will be analysed to determine reach, uptake and fidelity of the intervention delivery.

### Data management

Data collection forms will be identified by participant number only. Participant contact information and the code linking participant numbers to names will be stored in password-protected files on an encrypted USB key accessible only to AM. These files will be destroyed on completion of study publications. Completed data collection forms will be stored in a locked filing cabinet in a locked office and will be retained for 7 years post-publication as per University of Toronto procedures. Anonymized data will be entered into a spreadsheet stored on a different encrypted USB key before being transferred to statistical software for analysis.

### Statistical analysis

**Fig. 1:**
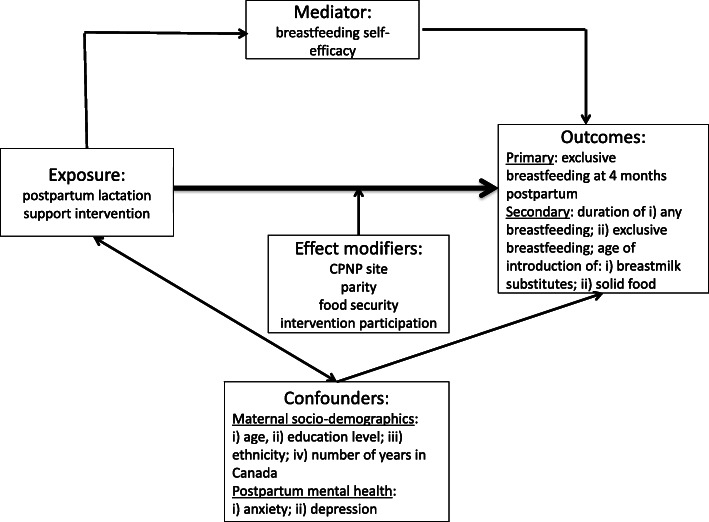
Conceptual model

Descriptive statistics will be generated for all variables. Primary and secondary outcome measures will be compared between the pre- and post-intervention groups using t-tests for continuous variables and chi-square statistics for categorical variables. Sub-group analysis will be performed to examine differences in outcomes based on the effect modifiers (program site, parity, food security status and intervention participation) as shown in the conceptual model (Fig. 1). Logistic regression analysis will be used to determine the relationship between breastfeeding self-efficacy scores at 2 months postpartum and breastfeeding practices at 4 and 6 months postpartum, controlling for confounders. All statistical analysis will be performed using IBM SPSS Statistics for Windows, version 26 (IBM Corp., Armonk, N.Y., USA).

## Discussion

The CPNP is a national initiative which aims to promote and support breastfeeding but currently lacks a formal framework for integrating postpartum lactation support, a key component of enabling women to achieve their breastfeeding goals. This pre/post intervention study aims to examine the effects on breastfeeding practices of pro-actively offering in-home lactation support to vulnerable women through two CPNP sites in Toronto. Feasibility will also be examined through analysis of monitoring data to determine the reach, uptake and fidelity of the intervention. We hypothesize that CPNP clients with access to our lactation support intervention will have higher breastfeeding self-efficacy scores at 2 months postpartum and be more likely to exclusively breastfeed their infants at 4 months postpartum compared with those in the pre-intervention group.

To date there has been limited evaluation of breastfeeding outcomes among CPNP participants. This study will contribute data on infant feeding practices at seven time points in the first 6 months postpartum, and will examine a range of covariates including measures of maternal mental health, household food insecurity and socio-demographic characteristics. Strengths of this research include the prospective data collection, theory-based intervention and inclusion of multiple covariates. AM and the Research Coordinator will attend the weekly program at both CPNP sites, and the Mandarin speaking Research Assistant will attend the weekly GST program. This embeddedness within the programs will contribute to the building of trust and rapport with participants, and is expected to result in high enrollment and retention rates [46]. However, the participant characteristics and infant feeding practices of women registered at the two study sites in Toronto may not be representative of CPNPs across the country and the pre/post study design is not as strong as a randomized trial for determining effectiveness. This research is therefore one step in the process of generating the evidence for appropriate models of integrating lactation support with the CPNP and thereby increasing breastfeeding, health and developmental outcomes for vulnerable infants in Canada.

## Data Availability

Not applicable.

## Abbreviations

CPNP: Canada Prenatal Nutrition Program
EPDS: Edinburgh Postnatal Depression Scale
GST: Great Start Together (perinatal program)
HB: Healthy Beginnings (perinatal program)
IBCLC: International Board Certified Lactation Consultant
5Ps: Parkdale Parents’ Primary Prevention Project
STAI: State-Trait Anxiety Inventory
