# Correction to: Effect on breastfeeding practices of providing in-home lactation support to vulnerable women through the Canada Prenatal Nutrition Program: protocol for a pre/post intervention study

**DOI:** 10.1186/s13006-021-00404-1

**Published:** 2021-07-29

**Authors:** Alison Mildon, Jane Francis, Stacia Stewart, Bronwyn Underhill, Yi Man Ng, Elle Richards, Christina Rousseau, Erica Di Ruggiero, Cindy-Lee Dennis, Deborah L. O’Connor, Daniel W. Sellen

**Affiliations:** 1 Nutritional Sciences, Temerty Faculty of Medicine, University of Toronto, Toronto, Ontario Canada; 2 Translational Medicine Program, The Hospital for Sick Children, Toronto, Ontario Canada; 3 Health Promotion and Community Engagement, Parkdale-Queen West Community Health Centre, Toronto, Ontario Canada; 4 The Stop Community Food Centre, Toronto, Ontario Canada; 5 Tamarack Institute, Waterloo, Ontario Canada; 6 Dalla Lana School of Public Health, University of Toronto, Toronto, Ontario Canada; 7 Lawrence-Bloomberg Faculty of Nursing, University of Toronto, Toronto, Ontario Canada; 8 Anthropology, Faculty of Arts and Sciences, University of Toronto, Toronto, Ontario Canada

**Correction to: Int Breastfeed J 16, 49 (2021)**

**https://doi.org/10.1186/s13006-021-00396-y**

Following publication of the original article [1], it was brought to our attention that the article had been published with an error in the References list: reference numbers 36 to 42 were out of order in the list.

The order of the reference list has now been corrected in the published article.
